# The Role of the SLC1A5 Transporter on Glutathione Homeostasis and Enterocyte Apoptosis in Chronic Treatment of Rats with Immunosuppressive Drugs

**DOI:** 10.3390/ijms26178330

**Published:** 2025-08-28

**Authors:** Tomasz Wawrowski, Anna Surówka, Michał Żołnierczuk, Piotr Prowans, Marta Grabowska, Patrycja Kupnicka, Marta Markowska, Mikołaj Kaczmarkiewicz, Weronika Sych, Edyta Zagrodnik, Karolina Kędzierska-Kapuza

**Affiliations:** 1 Department of Plastic, Endocrine and General Surgery, Pomeranian Medical University, 72-010 Szczecin, Poland; 2 Department of Vascular Surgery, General Surgery and Angiology, Pomeranian Medical University, 70-111 Szczecin, Poland; 3 Department of Histology and Developmental Biology, Faculty of Health Sciences, Pomeranian Medical University, 71-210 Szczecin, Poland; marta.grabowska@pum.edu.pl; 4 Department of Biochemistry and Medical Chemistry, Pomeranian Medical University, Powstańców Wlkp. 72, 70-111 Szczecin, Poland; 5 Clinical Department of Anesthesiology and Intensive Care of Adults and Children, Pomeranian Medical University, 72-010 Police, Poland; 6 Department of Gastroenterological Surgery and Transplantology, Centre of Postgraduate Medical Education in Warsaw, 137 Woloska St., 02-507 Warsaw, Poland

**Keywords:** immunosuppressive therapy, intestine, oxidative stress, SLC1A5, apoptosis, caspase, glutathione

## Abstract

Patients undergoing immunosuppressive therapy are at risk of adverse gastrointestinal symptoms such as diarrhea, nausea, intestinal barrier leakage, and nutrient malabsorption. One mechanism underlying these complications may be increased levels of oxidative stress in the cell, and thus an increased predisposition of enterocytes to programmed death. We examined the effects of triple immunosuppressive regimens on the concentration of glutathione, the SLC1A5 receptor, caspase-3, caspase-9, Bcl-2, Bax, and apoptosis in the rat intestine. For this purpose, we used Western blot analysis, ELISA, and the TUNEL method. The study began with 36 rats divided into six groups, which were administered the drugs for a period of six months. Our results suggest that chronic use of standard immunosuppressive regimens increases the risk of oxidative stress in the rat intestine, as manifested by increased expression of glutathione or the SLC1A5 transporter. The use of rapamycin in combination with cyclosporine A or mycophenolate mofetil leads to increased cellular apoptosis in the rat intestine, which is associated with a failure of compensatory mechanisms for elevated oxidative stress. The combination of tacrolimus with rapamycin results in the highest percentage of TUNEL positivity, and the apoptotic pathway is not a result of increased oxidative stress in the tissue.

## 1. Introduction

Organ transplantation in patients with end-stage renal, heart, or liver failure is one of the most important issues in modern medicine. For many patients, this form of treatment significantly prolongs survival and quality of life when other forms of treatment are no longer effective. In transplantology, the key condition for the survival of the transplanted organ is the effective suppression of the recipient’s immune response in order to prevent acute and chronic organ rejection with progressive loss of function [1]. Current immunosuppressive treatment regimens use combination therapies, most commonly involving calcineurin inhibitors (CNI) such as cyclosporine (CsA) or tacrolimus (TAC), mammalian target of rapamycin (mTOR) inhibitors such as rapamycin (RAP), antiproliferative drugs such as mycophenolate mofetil (MMF), and glucocorticosteroids (GCS). Their complementary immunosuppressive effects enable effective suppression of the recipient’s immune response, preventing episodes of organ damage in the graft rejection process while limiting the toxicity of individual drugs [2,3,4,5].

However, immunosuppressive therapy is associated with a number of organ complications, such as organ dysfunction, increased fibrosis, increased risk of cardiovascular events, and hyperglycemia [6]. Immunosuppressive drugs use presents negative consequences for the gastrointestinal tract as well. Complications such as diarrhea, nausea, intestinal barrier leakage, and malabsorption of nutrients have been observed [7]. One of the mechanisms underlying these complications may be increased oxidative stress and increased frequency of enterocyte apoptosis. Apoptosis plays an extremely important role in maintaining homeostasis in the body, contributing to the elimination of damaged cells, although its excessive activation can impair organ function and lead to progressive organ failure [8].

**Figure 1:**
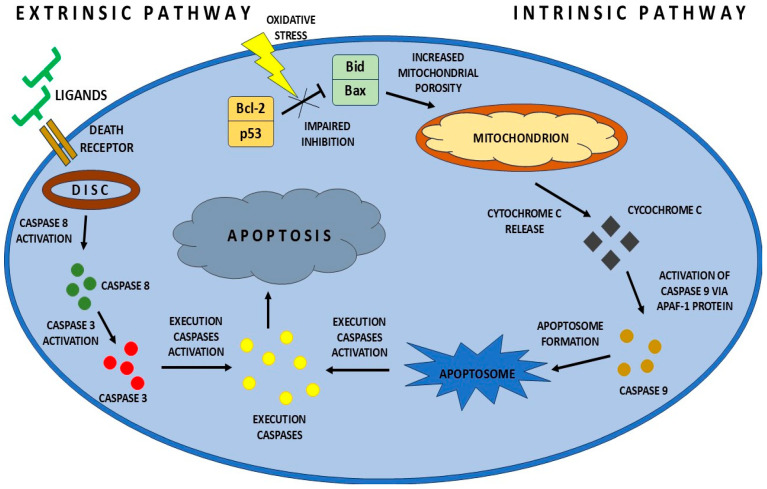
Simplified diagram of the intrinsic and extrinsic apoptosis pathway. In the intrinsic pathway, ligands activate the death receptor, leading to the formation of the death-inducing signaling complex (DISC). Caspase 8 and caspase 3 are activated, leading to the activation of executive caspases, which initiate the process of apoptosis. In the extrinsic pathway, the apoptosis-activating factor causes inhibition of Bid and Bax proteins by Bcl-2 and p53 proteins. This leads to an increase in mitochondrial porosity, which results in the release of cytochrome c and activation of caspase 9 by the Apaf-1 protein. As a result, an apoptosome is formed, which activates effector caspases, leading to apoptosis.

Apoptosis occurs mainly through two pathways: intrinsic and extrinsic. These pathways are regulated by the cascade activation of enzymes from the caspase family, which participate in the process of apoptosis. The intrinsic pathway is activated by increased oxidative stress in the cell, DNA damage, or the action of drugs. Pro-apoptotic Bax proteins are involved in the intrinsic activation of apoptosis, increasing the permeability of the mitochondrial membrane and thus the release of cytochrome c, which induces the intrinsic apoptosis process. Bcl-2 proteins, on the other hand, regulate the permeability of the mitochondrial membrane, acting anti-apoptotically. The release of cytochrome c from the mitochondria leads to the formation of the apoptosome complex and the activation of caspase-9, followed by caspase-3, which is an effector protein. The extrinsic pathway is initiated by death receptors. After ligand binding, they lead to the cascade activation of caspase-9, which directly or indirectly through the t-Bid protein activates the mitochondrial mechanism of apoptosis [9,10]. A simplified diagram of the intrinsic and extrinsic apoptosis pathway is shown in Figure 1.

**Figure 2:**
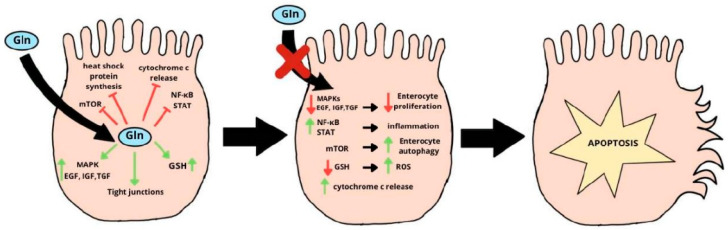
The mechanism of apoptosis activation resulting from impaired antioxidant function of glutathione. Gln—glutamine; GSH—glutathione; MAPK—mitogen-activated protein kinase; mTOR—mammalian target of rapamycin; EGF—epidermal growth factor; IGF—insulin-like growth factor; TGF—transforming growth factor; NF-κB—nuclear factor kappa-light-chain-enhancer of activated B cells; STAT—signal transducer and activator of transcription; ROS—reactive oxygen species.

However, there are protective mechanisms in the intestines that prevent excessive cell damage by protecting against oxidative stress. One of these is the synthesis of glutathione (GSH) from glutamine. GSH neutralizes reactive oxygen species (ROS), protecting intestinal cells from excessive damage and apoptosis. Under physiological conditions, the reduced form of glutathione predominates, and its deficiency, resulting from impaired access to its precursor, glutamine, leads to a decrease in cellular oxidative protection and activation of the intrinsic apoptosis pathway [11,12,13]. The diagram of intestinal cell damage caused by increased oxidative stress resulting from glutamine and glutathione deficiency is shown in Figure 2.

**Figure 3:**
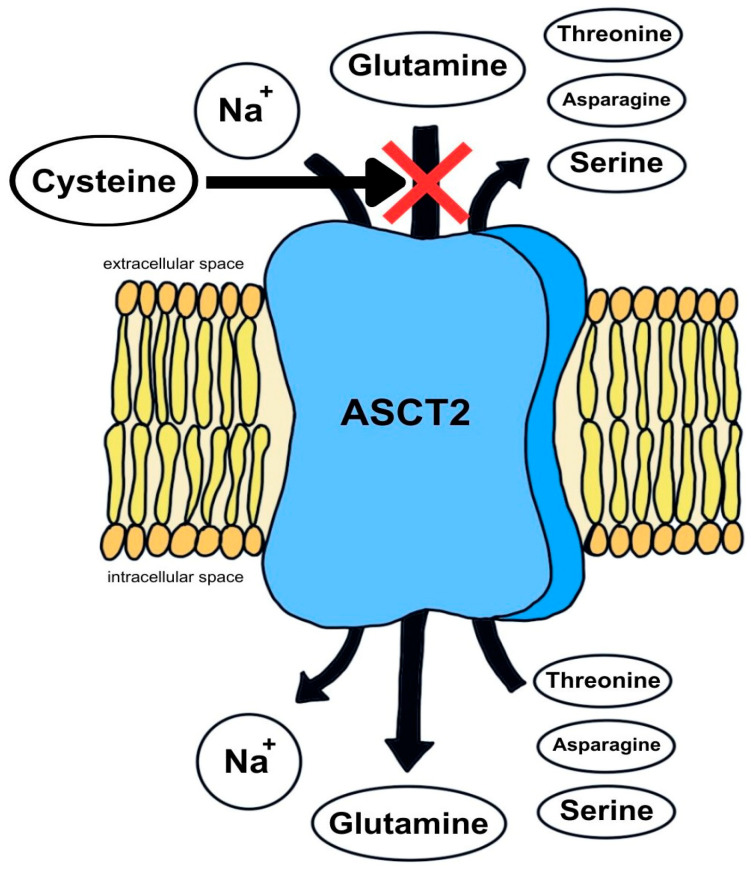
Schematic representation of the action of alanine, serine, and cysteine transporter type 2 (ASCT2). Na^+^—sodium cation.

Glutamine, as an amino acid, requires a specific transporter—alanine, serine, and cysteine transporter type 2 (ASCT2)—to enter the enterocyte. This transporter acts as an antiporter, exchanging extracellular glutamine for other neutral amino acids, mainly serine, asparagine, and threonine. Its activity increases under conditions of increased metabolic demand during stress, inflammation, or injury [14]. The function of the ASCT2 transporter is presented in Figure 3.

Fluctuations in glutamine levels have been observed during certain therapies, including immunosuppressive agents. It has been observed that the use of immunosuppressive drugs may lead to a decrease in glutamine levels, as well as impaired transport of glutamine in cells [15]. Unfortunately, there is lack of reports in the literature that clearly define the effect of immunosuppressive drugs belonging to the group of calcineurin inhibitors, mTOR inhibitors, antimetabolites, or glucocorticosteroids. In an experiment conducted by our research team, we attempted to determine how triple-drug immunosuppressive treatment regimens affect the ASCT2 transporter and glutathione concentration in cells, and thus the regulation of enterocyte apoptosis in animal models. This topic seems particularly important in the context of chronic oral immunosuppressive therapy, which increases the gastrointestinal tract’s exposure to the toxic effects of drugs. Investigating these relationships may lead to more accurate qualification of individual patients for specific treatment regimens, taking into account the recipient’s gastrointestinal disease burden or existing chronic intestinal diseases.

## 2. Results

### 2.1. Quantification of Apoptotic Cells Using the TUNEL Assay

**Figure 4:**
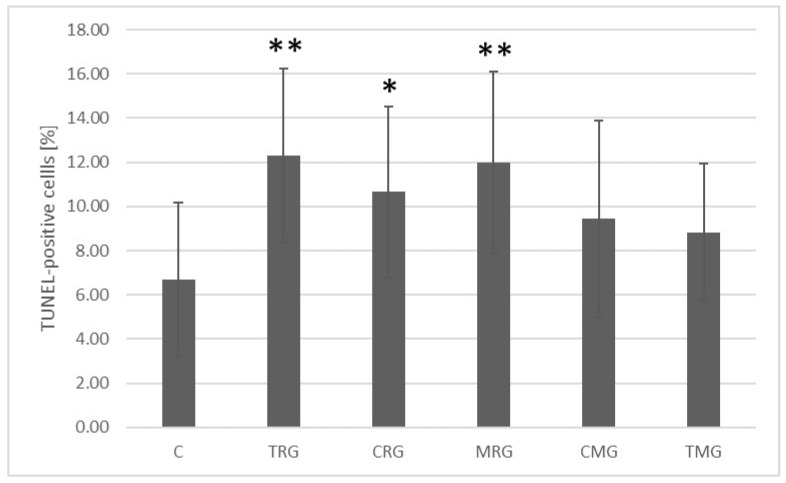
Comparison of the percentage of TUNEL-positive cells in the small intestine of rats in group C—control group; TRG—rats treated with tacrolimus, rapamycin, and glucocorticoid; CRG—rats treated with cyclosporine A, rapamycin, and glucocorticoid; MRG—rats treated with mycophenolate mofetil, rapamycin, and glucocorticoid; CMG—rats treated with cyclosporin A, mycophenolate mofetil, and glucocorticoid; TMG—rats treated with tacrolimus, mycophenolate mofetil, and glucocorticoid. Results are presented as medians and ranges. TUNEL—terminal deoxyribonucleotidyltransferase labeling of dUTP strand ends. * *p* < 0.05, ** *p* < 0.005, Mann–Whitney U test.

**Figure 5:**
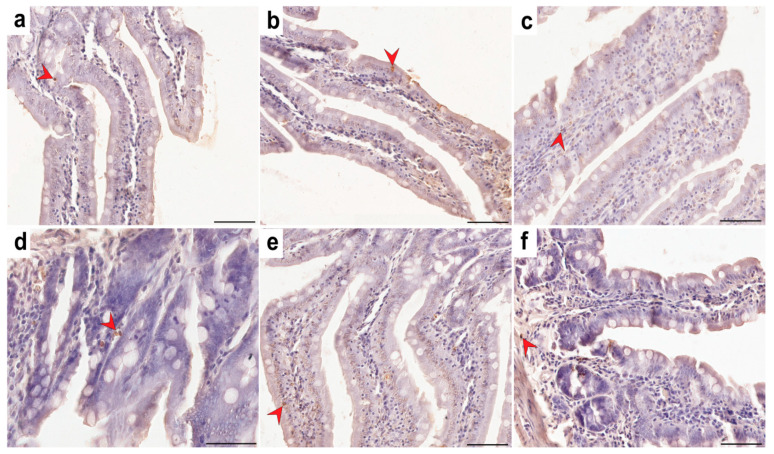
Representative light micrographs showing TUNEL-positive cells (red arrowheads) in the small intestine of rats in group C—control group (**a**); TRG—rats treated with tacrolimus, rapamycin, and glucocorticoid (**b**); CRG—rats treated with cyclosporine A, rapamycin, and glucocorticoid (**c**); MRG—rats treated with mycophenolate mofetil, rapamycin, and glucocorticoid (**d**); CMG—rats treated with cyclosporin A, mycophenolate mofetil, and glucocorticoid (**e**); TMG—rats treated with tacrolimus, mycophenolate mofetil, and glucocorticoid (**f**). Scale bar—50 µm.

In the control and experimental groups of rats, TUNEL-positive cells in the small intestine were characterized by nuclei stained brown. The percentage of TUNEL-positive cells in the TRG (*p* = 0.001), CRG (*p* = 0.014), and MRG (*p* = 0.001) groups was significantly higher than in the control group (Figure 4). Representative light micrographs showing TUNEL-positive cells are shown in Figure 5.

### 2.2. Quantitative Analysis of Caspase 3 Concentration

**Figure 6:**
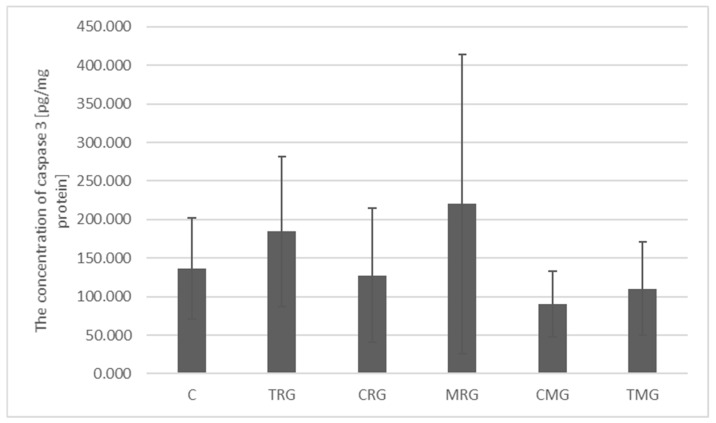
Expression of total caspase-3 protein measured by ELISA in the intestines of C rats—control group; TRG—rats treated with tacrolimus, rapamycin, and glucocorticoid; CRG—rats treated with cyclosporin A, rapamycin, and glucocorticoid; MRG—rats treated with mycophenolate mofetil, rapamycin, and glucocorticoid; CMG—rats treated with cyclosporin A, mycophenolate mofetil and glucocorticoid; TMG—rats treated with tacrolimus, mycophenolate mofetil and glucocorticoid. Data represent mean ± standard deviation. Mann–Whitney U test.

The concentration of caspase 3 in the control group was 136.78 pg/mg protein. Lower concentrations were observed in the CRG (127.53 pg/mg protein), CMG (78.91 pg/mg protein) and TMG (110.45 pg/mg protein) groups; however, they were not significantly different from the control group. Higher concentrations were characteristic of the TRG (184.77 pg/mg protein) and MRG (220.06 pg/mg protein) groups, but these changes were also not statistically significant (Figure 6).

### 2.3. Quantitative Analysis of Caspase 9 Concentration

**Figure 7:**
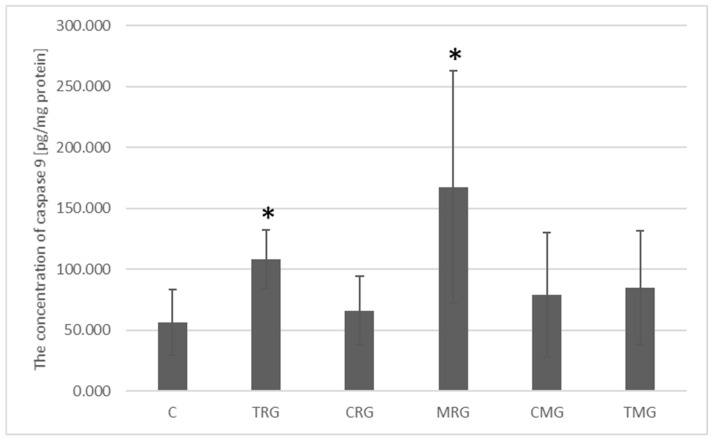
Expression of caspase 9 protein measured by ELISA in the intestines of rats from C—control group; TRG—rats treated with tacrolimus, rapamycin, and glucocorticoid; CRG—rats treated with cyclosporin A, rapamycin, and glucocorticoid; MRG—rats treated with mycophenolate mofetil, rapamycin, and glucocorticoid; CMG—rats treated with cyclosporin A, mycophenolate mofetil, and glucocorticoid; TMG—rats treated with tacrolimus, mycophenolate mofetil, and glucocorticoid. Data represent mean ± standard deviation. * *p* < 0.05, Mann–Whitney U test.

The highest concentration of caspase 9 protein was recorded in the MRG group. It amounted to 167.63 pg/mg protein and was significantly different from the control group’s protein concentration of 56.52 pg/mg protein (*p* = 0.009). A significantly higher concentration was also observed in the TRG group, where it reached an average of 108.20 pg/mg of protein and was 1.94 times higher than the concentration in the control group (*p* = 0.015). In the CRG, CMG and TMG groups, the average concentrations were 66.34 pg/mg protein, 78.91 pg/mg protein and 85.01 pg/mg protein, respectively, and were not significantly different from the average amount of caspase 9 protein in the control group (Figure 7).

### 2.4. Quantitative Analysis of Bcl-2 Protein Concentration

**Figure 8:**
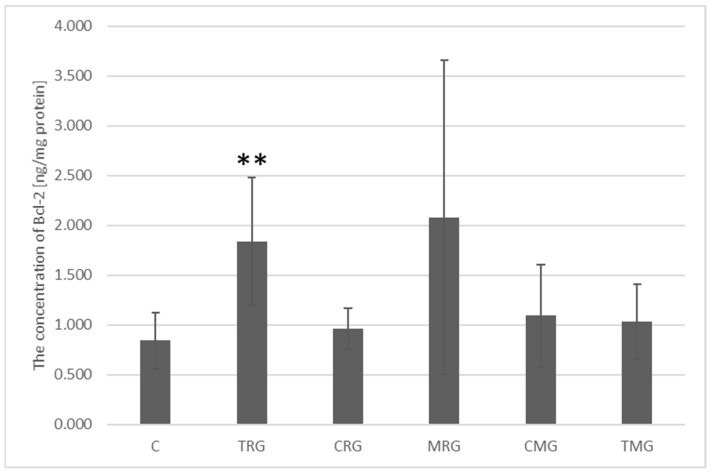
Bcl-2 protein expression measured by ELISA in the intestines of rats from C—control group; TRG—rats treated with tacrolimus, rapamycin, and glucocorticoid; CRG—rats treated with cyclosporin A, rapamycin, and glucocorticoid; MRG—rats treated with mycophenolate mofetil, rapamycin, and glucocorticoid; CMG—rats treated with cyclosporin A, mycophenolate mofetil, and glucocorticoid; TMG—rats treated with tacrolimus, mycophenolate mofetil, and glucocorticoid. Data represent mean ± standard deviation. ** *p* < 0.005, Mann–Whitney U test.

The highest Bcl-2 protein concentration was observed in the MRG group and was 2.083 ng/mg protein; however, this result was not significantly different from the Bcl-2 concentration in the control group, which was 0.842 ng/mg protein. In the TRG group, the Bcl-2 protein concentration was 1.863 ng/mg protein. This value was significantly different from the control group (*p* = 0.004). The other groups were not significantly different from the control group, and the Bcl-2 concentration in them was: CRG = 0.964 ng/mg protein, CMG = 1.094 ng/mg protein and TMG = 1.032 ng/mg protein (Figure 8).

### 2.5. Quantitative Analysis of Bax Protein Concentration

**Figure 9:**
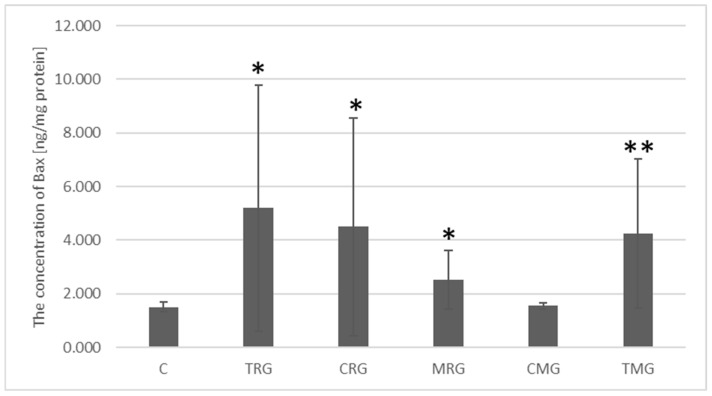
Bax protein expression measured by ELISA in the intestines of rats with C—control group; TRG—rats treated with tacrolimus, rapamycin, and glucocorticoid; CRG—rats treated with cyclosporin A, rapamycin, and glucocorticoid; MRG—rats treated with mycophenolate mofetil, rapamycin, and glucocorticoid; CMG—rats treated with cyclosporin A, mycophenolate mofetil, and glucocorticoid; TMG—rats treated with tacrolimus, mycophenolate mofetil, and glucocorticoid. Data represent mean ± standard deviation. * *p* < 0.05, ** *p* < 0.005, Mann–Whitney U test.

The concentration of Bax protein in the control group was 1.511 ng/mg protein. In the four study groups, the concentration of this protein was significantly higher than in the control group. In the TRG group it was 3.44 times higher (*p* = 0.03), in the CRG group it was 2.98 times higher (*p* = 0.04), in the MRG group Bax protein concentration increased 1.68 times (*p* = 0.04), and in the TMG group it increased 2.82 times (*p* = 0.002). In the CMG group, Bax protein concentration was 1.03 ng/mg and was not significantly different from the control group (Figure 9).

### 2.6. Quantitative Analysis of SLC1A5 (ASCT2) Transporter Expression

**Figure 10:**
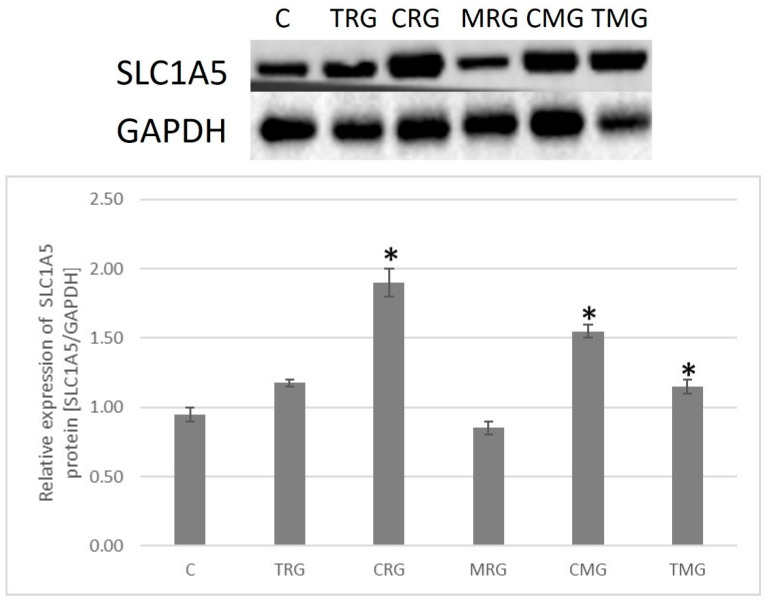
Representative Western blot and graph showing relative expression of SLC1A5 protein in the intestines of rats, C—control group; TRG—rats treated with tacrolimus, rapamycin, and glucocorticoid; CRG—rats treated with cyclosporine A, rapamycin, and glucocorticoid; MRG—rats treated with mycophenolate mofetil, rapamycin, and glucocorticoid; CMG—rats treated with cyclosporin A, mycophenolate mofetil, and glucocorticoid; TMG—rats treated with tacrolimus, mycophenolate mofetil, and glucocorticoid. Data represent mean ± standard deviation. * *p* < 0.05, Mann–Whitney U test.

The highest mean expression of the SLC1A5 transporter was in the intestinal tissue of the CRG group. It was significantly different from the control group (*p* = 0.046). Significant differences were also seen in the CMG and TMG groups (*p* = 0.046). The lowest expression of the transporter was observed in the MRG group (Figure 10).

### 2.7. Quantitative Analysis of Glutathione Concentration

**Figure 11:**
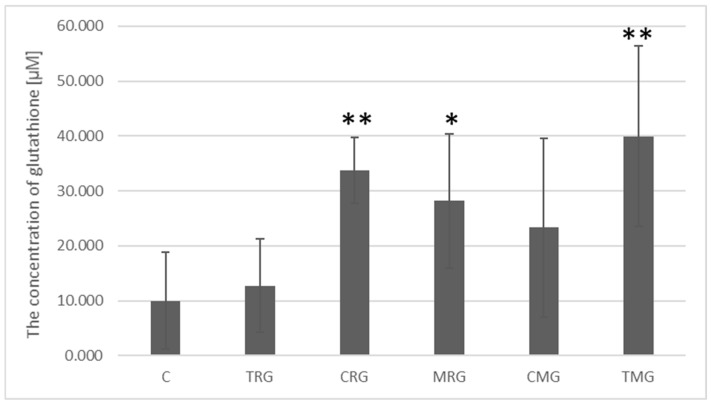
Glutathione concentration in the intestines of rats measured by ELISA in C—control group; TRG—rats treated with tacrolimus, rapamycin, and glucocorticoid; CRG—rats treated with cyclosporin A, rapamycin, and glucocorticoid; MRG—rats treated with mycophenolate mofetil, rapamycin, and glucocorticoid; CMG—rats treated with cyclosporin A, mycophenolate mofetil and glucocorticoid; TMG—rats treated with tacrolimus, mycophenolate mofetil, and glucocorticoid. Data represent mean ± standard deviation. * *p* < 0.05, ** *p* < 0.005, Mann–Whitney U test.

Glutathione concentrations in the control group averaged 9.98 µM. Significantly higher glutathione concentrations were present in the intestinal tissue of animals from the CRG groups, where the increase was 3.38-fold (*p* = 0.002), MRG, where a 2.82-fold increase was noted (*p*- = 0.03), and in the TMG group, where the increase was the highest (4-fold) (*p* = 0.009). Higher glutathione concentrations were also noted in the CMG and TRG groups relative to the control group, but the differences were not statistically significant (Figure 11).

## 3. Discussion

The results obtained in our study revealed complex interrelationships between apoptosis, oxidative stress, and glutamine metabolism in rats treated with multi-drug immunosuppressive regimens. Apoptosis is a physiological process of programmed cell death in response to abnormal signals from the external environment or a defect in the structure and function of the cell itself. Unlike necrosis, which is a chaotic process of eliminating mechanically damaged cells, apoptosis occurs in a controlled and organized manner without a generalized inflammatory process. During programmed cell death, the cell shrinks, chromatin condenses, DNA fragments, and apoptotic bodies are formed, which are then neutralized by phagocytic cells. Apoptosis, which allows for the elimination of damaged cells, plays a significant role in numerous biological processes, controls the body’s immune response, and, above all, influences the maintenance of proper tissue homeostasis [10,16,17].

The extrinsic pathway of apoptosis, also known as the receptor pathway, is activated by signals acting on death receptors FasR (Fas Receptor), TNFR1 (Tumor Necrosis Factor Receptor 1) located on the cell surface. The binding of a ligand (FasL or TNF-α) to the receptor causes a change in its conformation and activation of the cytoplasmic domain of the receptor, the death domain (DD). The death domain binds adaptor proteins such as FADD (Fas-Associated Death Domain). FADD binds procaspase-8 or procaspase-10 via the death effector domain (DED), forming the DISC (Death-Inducing Signaling Complex). In the DISC complex, caspase-8 (or caspase-10) is autoproteolytically activated, which in turn activates caspase-3, the main effector caspase, leading to protein degradation and ultimate cell death [10,17,18].

The intrinsic apoptosis pathway is based on processes occurring in the mitochondria. As a result of an increase in reactive oxygen species, oxidative stress, calcium ion concentration, certain cytokines, the action of pathogens, or the influence of physical factors, the process of apoptosis is initiated. The P53 protein is translocated from the cytoplasm to the mitochondria, where it forms a complex with the anti-apoptotic proteins Bcl-2/Bcl-Xl, thereby blocking their action. The proapoptotic Bax protein undergoes oligomerization in the mitochondrial membrane, which causes the opening of mitochondrial channels and the release of cytochrome c. In the cytoplasm, cytochrome c, together with the Apaf-1 factor and procaspase-9, forms an apoptosome. Activation of the apoptosome leads to the initiation of the executive caspase cascade and cell death [19,20,21].

The endoplasmic reticulum apoptosis pathway is one of the alternative apoptosis pathways in which cell death is initiated by endoplasmic reticulum (ER) stress. An increase in ion concentration, especially calcium ions, and the accumulation of abnormal proteins in the cytoplasm leads to the blocking of protein transport to the ER. Caspase 12, located in the endoplasmic reticulum membrane, activates the executive caspases, caspase 7 and caspase 8, which lead to apoptosis. The reticulum pathway can be initiated spontaneously or can interact with the receptor or mitochondrial pathways. Programmed cell death involves caspases, but an alternative pathway without proteases has also been reported. A stress factor triggers the release of calcium from the endoplasmic reticulum, which can bind to many proteins, including calpain. This enzyme activates the proapoptotic protein Bax, which migrates to the mitochondria, causing the release of cytochrome c and inducing apoptosis [22,23].

The results of our experiment show that all three-drug immunosuppressive treatment regimens caused increases in the number of TUNEL-positive cells, but only in the TRG, CRG, and MRG groups the values obtained were statistically significant. In order to confirm the occurrence of apoptosis, we analyzed two caspases, caspase-3 and caspase-9, and two proteins from the Bcl-2 family, Bcl-2 and Bax. Caspase-3 expression was elevated in the TRG and MRG groups, but none of the values obtained were statistically significant. A statistically significant increase in caspase-9 expression was observed in the TRG and MRG groups. The measurement of the proapoptotic protein Bax was significantly higher in the TRG, CRG, MRG, and TMG groups, with the highest values recorded in the TRG group. An increase in the expression of the antiapoptotic protein Bcl-2 was also observed in this group. The above results indicate the predominance of the intrinsic apoptosis pathway when using treatment regimens that induce a statistically significant increase in apoptotic cells in the TUNEL assay. Increased caspase 9 activity was observed in the TRG and MRG groups. The lack of a statistically significant increase in caspase-3 levels in all groups showing an elevated proportion of TUNEL-positive cells may result from high protein degradation during intense apoptosis or may reflect the activation of alternative apoptotic pathways, such as the endoplasmic reticulum (ER) stress-related pathway. Caspases may be activated in the ER pathway, with calcium ions and Bcl-2 family proteins playing a key role. Both the intrinsic apoptotic pathway and the endoplasmic reticulum pathway can be activated during oxidative stress. In the TRG group, an increase in the expression of the anti-apoptotic protein Bcl-2 was also observed. Under pathological conditions, the simultaneous increase in pro- and anti-apoptotic proteins reflects the active regulation of programmed cell death, and the Bax/Bcl-2 ratio is of key importance for survival. In the TRG group, the balance shifted towards the proapoptotic protein Bax, resulting in a significant increase in TUNEL-positive cells.

Immunosuppressive treatment exert a significant influence on the development of oxidative stress, which manifests itself in increased levels of lipid peroxidation markers and reduced activity of antioxidant enzymes [24,25,26]. Patients treated with calcineurin inhibitors show higher levels of oxidative stress compared to healthy individuals, while taking cyclosporine A, rapamycin, or mycophenolate mofetil is associated with severe endothelial oxidative stress [27,28,29]. The presence of free radicals and oxidative stress are considered to be significant factors influencing the development of serious complications of immunosuppressive treatment, including generalized inflammation and significant organ damage [24].

Our study highlights the importance of glutamine (Gln) metabolism in protecting cells from apoptosis caused by oxidative stress. Glutamine inhibits the intrinsic apoptosis pathway by reducing the release of cytochrome C from mitochondria and also protects the cell from damage by limiting the synthesis of heat shock proteins. This amino acid is involved in the synthesis of glutathione (GSH), which is one of the main neutralizers of reactive oxygen species (ROS). Under normal conditions, the concentration of the reduced form of glutathione (GSH) is significantly higher than that of the oxidized form (GSSG). Increased oxidative stress causes a decrease in GSH and requires the supply of a precursor in the form of glutamine. Abnormalities in glutathione formation reduce the antioxidant protection of cells and can lead to increased apoptosis of enterocytes [30,31,32]. In our study, we measured total glutathione concentration, as the reduced form of GSH is highly susceptible to oxidation already at the stage of isolation and sample preparation, which could lead to falsification of results. The body’s demand for Gln increases significantly in states of inflammation, stress, or as a result of injury. Deficiencies are supplemented with exogenous Gln, which is absorbed from the intestines along with food. The intestinal transporter SLC1A5 (ASCT2) plays a significant role in maintaining normal glutamine concentrations in the organism [33,34].

The CMG and TMG groups showed increased expression of SLC1A5, and the TMG group also reported elevated glutathione levels. No statistically significant increase in the number of TUNEL-positive cells was observed in either group. The results suggest that the use of calcineurin inhibitors in combination with mycophenolate mofetil did not cause significant cell damage, and the mechanisms counteracting excessive stress did not become decompensated. In all groups treated with rapamycin, a significant increase in apoptosis was observed in rat intestinal cells. In the CRG and MRG groups, an increase in glutathione levels was observed, but in the CRG group, SLC1A expression was also elevated. Higher glutathione values indicate ongoing oxidative stress, while in the CRG group, compensatory mechanisms in the form of increased glutamine uptake were preserved. In the MRG group, the deficiency of glutathione precursors resulted in a higher rate of TUNEL-positive cell. The TRG group was the only one that did not show significantly increased expression of either the SLC1A5 transporter or glutathione, with the highest rate of intestinal cell apoptosis among all tested groups.

The observations made may indicate a factor other than oxidative stress triggering programmed death of intestinal cells. There are no publications in the available literature on the negative impact of immunosuppressive treatment on enterocytes, but this is a very important topic, especially since these drugs are also widely used in the treatment of autoimmune diseases of the intestine. In the context of organ recipients, it should be noted that the use of treatment regimens based on rapamycin may be associated with increased toxic effects of drugs on the intestinal epithelium. The occurrence of adverse gastrointestinal symptoms during this treatment should prompt consideration of the continuation of the selected regimen, especially in patients with intestinal diseases. The presented results are a prelude to further research on the effect of chronic immunosuppressive treatment on the intestines, which seems reasonable, especially from the perspective that all drugs are administered orally. Gaining such knowledge will minimize the risk of adverse effects or help counteract changes in the intestinal epithelium by taking exogenous glutamine in the daily diet of patients undergoing immunosuppressive treatment.

## 4. Material and Methods

### 4.1. Material

This study was carried out on tissue material in the form of the intestines of Wistar rats, which was collected and secured from the Department of Nephrology, Transplantology and Internal Medicine at the Pomeranian Medical University in Szczecin. The biological material was obtained as part of an experiment carried out by Kedzierska et al. [35]. The study was approved by the Local Ethical Committee for Animal Experiments (decision number 24/2008, dated 24 November 2008).

**Figure 12:**
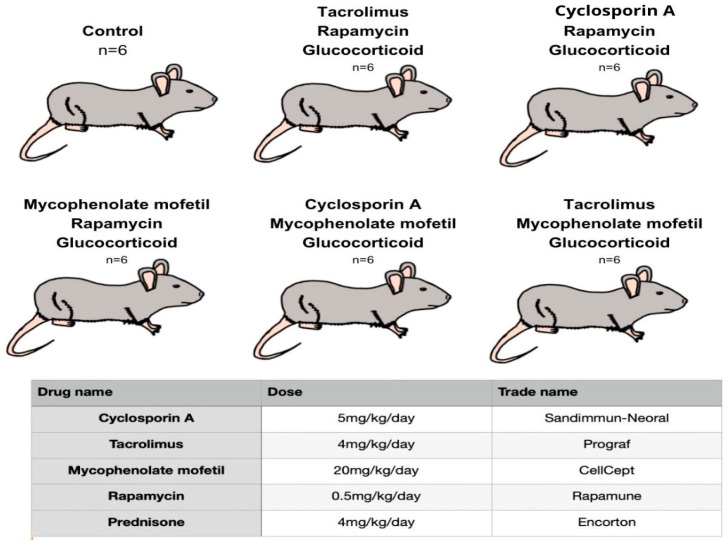
Types of three-drug immunosuppressive treatment regimens and doses of drugs used in the study.

The study was conducted on 36 male rats aged 14 weeks. The average weight of the individuals was 305 g. The animals were purchased from a licensed breeder and then transported to the animal house of the Pomeranian Medical University in Szczecin. The rats were divided into 6 groups and placed equally in 6 cages each. The air temperature in the animal room was 22 °C, humidity reached 55%, and the light day was divided 12/12. All rats experienced a two-week adaptation period, during which they received LSM laboratory diet (17.6% protein, 1474 kJ/100 g) and water. Five groups of subjects were exposed to immunosuppressive drugs and one untreated control group. Animals in each of the 5 groups were then given an oral form of one of the three-drug immunosuppressive regimens once daily (Figure 12). The control group was not treated with any drug. The drug doses used allowed the therapeutic concentrations of each substance to be reached in the blood.

After 3 months, the animals were weighed, and drug doses were adjusted to the weight result. Two individuals in the CRG group died before the end of the experiment. Six months after the start of the experiment, the rats were anesthetized with intraperitoneal administration of ketamine at a dose of 50 mg/kg body weight. The animals were dissected, and tissue material in the form of intestinal fragments was collected. Samples from each rat were placed in a vat of liquid nitrogen and then preserved in a low-temperature freezer (−86 °C). The remaining organ fragments were fixed in 4% paraformaldehyde, then embedded in paraffin blocks.

### 4.2. Methods

#### 4.2.1. Quantitative Analysis of TUNEL-Positive Cells

The TUNEL (terminal deoxynucleotidyl transferase dUTP nick-end labeling) assay was performed according to the manufacturer’s guidelines (ApopTag^®^ Peroxidase in situ Apoptosis Detection Kit; Milli- pore, Billerica, MA, USA) to detect nuclear DNA fragmentation associated with apoptosis. Intestinal fragments were deparaffinized, rehydrated, and digested with proteinase K (Dako, Glostrup, Denmark). Endogenous peroxidase activity was blocked by applying peroxidase blocking solution (Dako, Glostrup, Denmark). Slides were then incubated with terminal deoxynucleotidyl transferase (TdT; Millipore, Billerica, MA, USA) for 60 min in a humid chamber at 37 °C. Subsequently, the slides were incubated with peroxidase-conjugated anti-digoxigenin antibody for 30 min in a humid chamber. To visualize the reaction, diaminobenzidine (DAB; Dako, Glostrup, Denmark) was used.

The sections were subjected to Mayer’s hematoxylin contrast staining, then dehydrated and covered with a coverslip. The scrapings were examined under a light microscope (Olympus BX 41, Hamburg, Germany). Slides with TUNEL immuno-stained intestinal sections were scanned at 200× magnification (0.25 µm/pixel resolution) using a ScanScope AT2 scanner (Leica Mi- crosystems, Wetzlar, Germany).

The obtained digital images were analyzed on a computer screen using ImageScope viewer (version 11.2.0.780; Aperio Technologies, Vista, CA, USA). The v9 nuclear algorithm (version 9.1; Aperio Technologies, Vista, CA, USA) was used for quantitative analysis of TUNEL-positive cells. Areas of analysis were determined manually. The percentage of TUNEL-positive nuclei was calculated in 30 random fields for each group.

#### 4.2.2. Protein Isolation for ELISA and Western Blot Assays

The tissues were subjected to hammer homogenization in liquid nitrogen. RIPA lysis buffer (cat. 89901, Thermo Scientific, Pierce Biotechnology, Waltham, MA, USA) containing phosphatase and protease inhibitors (cOmplete™, Mini Protease Inhibitor Cocktail, Roche, Switzerland, PhosSTOP™, Roche, Switzerland) was then added to each sample. Tissues were incubated on ice for 20 min and then centrifuged (14,000× *g* for 20 min). Protein concentration was determined in the collected supernatant. The samples were stored at −80 °C until further analysis.

Determination of the protein concentration in the samples was performed by the modified biuret and Lowry’s bicinchoninic acid (BCA) method using a commercial Micro BCA Protein Assay Kit (Thermo Scientific, Rockford, IL, USA). The method uses the reduction of Cu^2+^ ions in alkaline medium by proteins to Cu^+^. BCA, in turn, forms a colored complex with Cu^+^. The concentration of this complex is measured spectrophotometrically with absorbance at 562 nm. The modified method is more sensitive than the classical Lowry method.

Samples were mixed with reagents according to the manufacturer’s instructions and incubated at 37 °C for 2 h. After this time, the absorbance of the samples was measured using a Lambda 40 spectrophotometer (Perkin Elmer, Waltham, MA, USA). At the same time, a standard curve was prepared using the albumin standard included in the reagent kit. The protein concentration of the samples was determined based on the obtained curve. All samples were analyzed twice, and the measurement result is the average of the two measurements.

#### 4.2.3. ELISA Immunoenzymatic Test: Determination of Protein Concentrations of Bcl-2, Bax, Caspase 3 and Caspase 9

The concentration of Bcl-2, Bax, caspase 3, and caspase 9 proteins was performed using commercially available ELISAs according to the manufacturer’s instructions (cat. ER0762, ER0512, ER0143, ER0804, respectively, FineTest, Wuhan, China).

Standards, a blank sample, and appropriately diluted samples were added to the plates in consecutive wells. All samples were analyzed in duplicate. Plates were incubated at 37 °C, washed, and then a biotinylated antibody detecting the antigen in question was added to form a complex with the antibody on the microplate. The plates were incubated at 37 °C, washed, and then incubated with horseradish peroxidase (HRP)-streptavidin conjugate. Streptavidin has an extremely high binding affinity for biotin, ensuring the rapid formation of a specific and stable bond. After incubation, a solution of the substrate 3, 3′, 5, 5′-tetramethylbenzidine (TMB) was added to each well. After color development, the absorbance was measured spectrophotometrically at 450 nm. The concentration of Bcl-2, Bax, caspase 3, and caspase 9 in the samples was determined by comparing the absorbance of the samples with that of the curve prepared according to the instructions.

#### 4.2.4. Western Blot Measurement of SLC5A1 Protein Expression

Samples for electrophoretic separation were prepared by diluting the protein with protease and phosphatase inhibitor-containing buffer (cOmplete™, Mini Protease Inhibitor Cocktail, Roche, Basel, Switzerland, PhosSTOP™, Roche, Basel, Switzerland) to which a fourth part of loading buffer (Bio-Rad, Feldkirchen, Germany) containing a tenth of β-mercaptoethanol was added. The sample with the loading solution was heated at 90 °C for 7 min in a Compact Thermomixer (Eppendorf, Warsaw, Poland) to denature the proteins. After the samples cooled, the mixture was transferred to a SurePAGE™ gradient gel (cat. GSM00660, GenSignal, Poznan, Poland) and electrophoretic separation of the proteins was performed.

The polyacrylamide gel prepared in this way was immersed in Tris-MOPS-SDS electrophoresis buffer (cat. M00138, GenSignal, Poznan, Poland), the tank was refilled, and electrophoretic separation was carried out at 180 V for 30 min. After electrophoresis was completed, protein electrotransfer was performed onto a 0.2 µm PVDF (polyvinylidene fluoride) membrane (Thermo Fisher Scientific™, Waltham, MA, USA) by wet transfer in commercial buffer (cat. M00139, GenSignal, Poznan, Poland) with 20% methanol at 70 V for 60 min.

Before incubation with the antibody, the membranes were placed in blocking buffer −5% skimmed milk for 60 min. Membranes were washed in neutral buffer. Protein expression was detected using an antibody against SLC1A5 (A-12054, Antibodies.com (4.04.2025), Stockholm, Sweden) diluted 1:500 and a secondary anti-rabbit H&L antibody (cat. ab205718, Abcam, Cambridge, UK). At the same time, expression of the reference protein, GAPDH (ab181602, Abcam, Cambridge, UK), was detected. To visualize the results, membranes were developed using an ECL (enhanced chemiluminescence) Advance Western Blotting Detection Kit (GE Healthcare, Chicago, IL, USA) and a Molecular Imager ChemiDock XRS+ instrument (Bio-Rad, Hercules, CA, USA). Densitometric analysis was performed using Image Lab Software 6.1 (Bio-Rad, Hercules, CA, USA).

#### 4.2.5. Determination of Glutathione Concentration by Enzymatic Method

Glutathione concentration in the samples was measured using a commercial kit (cat. MAK440, Sigma-Aldrich, Poland). After cell lysis and centrifugation, the supernatant was subjected to deproteinization. The procedure began with fresh preparation of a 5% solution of meta-phosphoric acid. This reagent was then added to the sample. The next step was to centrifuge the samples at 14,000× *g* for 5 min. The neutralized samples were placed in separate wells of a transparent 96-well plate. At the same time, a standard curve was prepared according to the manufacturer’s instructions, which was also placed on the plate. A working reagent consisting of buffer, glutathione reductase enzyme, NADPH and DTNB was added to the samples. The optical density of the mixture was tested immediately and after 10 min of incubation at 420 nm. The results obtained were calculated according to the formula GSH total (µM) = (Δ absorbance of sample − Δ absorbance of blank sample) × DF factor/slope of curve. All measurements were performed twice, and the results presented are the average of the measurements.

#### 4.2.6. Statistical Analysis

Statistical analyses for the TUNEL test were performed using TIBCO Statistica version 13.3 software (TIBCO Software Inc., Palo Alto, CA, USA). Arithmetic means (X), standard deviations (SD), medians (Me) and range were calculated. To assess differences between groups, the non-parametric Kruskal–Wallis test with Dunn’s multiple comparisons test was used for post hoc analysis. The level of statistical significance was *p* < 0.05. The results of ELISA, Western blot, and enzyme assay were statistically analyzed using Statistica 13 software and presented, as mean ± SD values. The Shapiro–Wilk W test did not show agreement with a normal distribution; the non-parametric Mann- Whitney U test was used to compare groups. Statistical significance was set at *p* < 0.05.

## 5. Conclusions

This study shows that chronic use of standard immunosuppressive treatment regimens increases the risk of oxidative stress in the intestines of rats, which manifests itself in increased expression of glutathione or the SLC1A5 transporter. The use of rapamycin in combination with cyclosporine A or mycophenolate mofetil leads to increased cell apoptosis in the intestines of rats, which is associated with the failure of compensatory mechanisms and increased oxidative stress. The combination of tacrolimus and rapamycin causes the highest percentage of TUNEL-positive cells, and the apoptosis pathway is not the result of increased oxidative stress in the tissue.

## Data Availability

Data supporting the findings of this study are available upon request from the corresponding author.
